# Traffic-Related Air Pollution and Stress: Effects on Asthma

**DOI:** 10.1289/ehp.11863

**Published:** 2008-09

**Authors:** Jane E. Clougherty, Laura D. Kubzansky

**Affiliations:** Harvard School of Public Health, Boston, Massachusetts, E-mail: jcloughe@hsph.harvard.edu

Chen et al. (2008) examined the potential for social stressors to influence responsiveness to environmental pollution. Contrary to their initial hypothesis, and to results we reported previously (Clougherty et al. 2007), their findings indicated that chronic stress was associated with asthma symptoms and heightened inflammatory profiles only in low nitrogen dioxide areas. We would like to note several key issues in the emerging research on social susceptibility to environmental pollutants that should be considered as research on this work moves forward.

One key issue is that the relative timing of psychosocial stressors and physical exposures, which Chen et al. (2008) did not present, is critical for at least two reasons:

- Acute and chronic stress produce substantively different physiologic sequelae. Acute stress can induce bronchodilation with elevated cortisol (possibly masking short-term detrimental respiratory effects of pollution), whereas chronic stress can result in cumulative wear and tear (allostatic load) and suppressed immune function over time, increasing general susceptibility (McEwen and Seeman 1999).
- Temporal relationships between stress and pollution exposures matter. Depending on when measures are obtained, exposure misclassification is possible, which may influence the directionality of observed interactions. Chen et al. (2008) stated that the measured 6-month stress and NO_2_ periods do not overlap, but they did not specify whether the stress measure preceded the 1998–2003 NO_2_ exposure window or the amount of time that passed between exposures. If the stress interval occurred first, some increased susceptibility to subsequent pollution is plausible, provided that chronic stress effects predominate over acute effects. If, however, the stress interval occurred after NO_2_ exposures, the interaction is potentially problematic, because we must then assume that stress levels measured after the 6-year NO_2_ period (1998–2003) are relevant for the earlier time, which may not be the case. If, for example, respondents compared current stress to prior experience, an individual reporting high stress for one interval may have experienced lower stress previously, during those “reference” periods corresponding to the NO_2_ window—potentially producing a negative interaction, as Chen et al. (2008) observed. More broadly, careful attention to relative timing and durations of stress and pollution exposures is critical in maintaining directionality and interpretability as we progress with this research.

Second, Chen et al.’s finding of significant effects of stress only in low-NO_2_ areas (Chen et al. 2008) points to the possibility of nonlinear interactions and saturation effects at high exposures. Similarly, our group (Clougherty et al. 2006) reported that asthmatic children of families reporting higher fear of violence showed less symptom improvement in response to allergen-reducing indoor environmental interventions. Our results, counter to our initial hypotheses, suggested a saturation effect in our very high-exposure public housing cohort, where either high exposure alone may have been adequate to induce or maintain symptoms.

Third, Chen et al. (2008) did not address the spatial covariance among stress, socioeconomic status, and pollution, which can confound geographic information system–based air pollution epidemiology. In particular, communities near highways, with higher traffic-related pollution and lower property values, may be disproportionately composed of families having lower socioeconomic status. Because of this potential for spatial autocorrelation and thus confounding, accurate fine-scale exposure measurement is critical. However, Chen et al. (2008) did not present pollution or stress maps, the NO_2_ model was not formally validated to this cohort’s specific spatial characteristics, and spatial patterns in stress were not explored; thus we are left wondering whether, and how, spatial misclassification and confounding may be at play. Relatedly, social–physical correlations may vary by geographic scale (e.g., across vs. within neighborhoods); although a given neighborhood may have high mean pollution and stress, it is harder to argue that particular individuals (or residences) within these neighborhoods would be relatively more exposed to both (i.e., individuals living closer to highways are not necessarily more exposed to violence or family stress than are other community members).

Fourth, Chen et al. (2008) reported results for 73 asthmatic children. However, in the absence of information on disease chronicity, severity, or adequacy of medical treatment, it may be difficult to truly assess the influence of either stress or traffic-related pollution. Relatedly, it is important to distinguish between processes related to illness onset from those related to progression or exacerbation, and whether the negative interaction observed in their study could be expected in healthy adolescents.

Finally, the cohort studied by Chen et al. (2008) varied considerably in age (9–18 years), but the authors did not consider age-related asthma characteristics and responsiveness to family stressors and air pollution. Age stratification should have been used to compare the strength of individual and combined effects at multiple ages. It would also be interesting to know whether non–family-related stressors would produce similar interactions at all ages.

The issues we have highlighted—temporal relationships between stressors and pollution, nonlinearity and saturation effects, spatial correlations, age-related susceptibility, and distinctions between illness etiology and exacerbation—will be critical in the further study of social–environmental interactions. These effects may distort observed associations (e.g., saturation effects may reverse interactions at high exposures), but with sustained attention to these issues, we can better understand joint effects of social and physical environments on health.
